# Signatures of adaptation to plant parasitism in nematode genomes

**DOI:** 10.1017/S0031182013002163

**Published:** 2014-01-30

**Authors:** DAVID McK. BIRD, JOHN T. JONES, CHARLES H. OPPERMAN, TAISEI KIKUCHI, ETIENNE G. J. DANCHIN

**Affiliations:** 1 Bioinformatics Research Center, NC State Univ, Raleigh, NC 27695, USA; 2 Plant Nematode Genetics Group, Dept. of Plant Pathology, NC State Univ, Raleigh, NC 27695, USA; 3 James Hutton Institute, Invergowrie, Dundee DD2 5DA, UK; 4 Division of Parasitology, Faculty of Medicine, University of Miyazaki, Miyazaki 889-1692, Japan; 5 Forestry and Forest Products Research Institute, Tsukuba 305-8687, Japan; 6 INRA, UMR 1355, Institut Sophia Agrobiotech, F-06903, Sophia-Antipolis, France; 7 Université de Nice Sophia-Antipolis, Institut Sophia Agrobiotech, F-06903, Sophia-Antipolis, France; 8 CNRS, UMR 7254, Institut Sophia Agrobiotech, F-06903, Sophia-Antipolis, France

**Keywords:** nematodes, genomes, plant parasitism, adaptation, convergence

## Abstract

Plant-parasitic nematodes cause considerable damage to global agriculture. The ability to parasitize plants is a derived character that appears to have independently emerged several times in the phylum Nematoda. Morphological convergence to feeding style has been observed, but whether this is emergent from molecular convergence is less obvious. To address this, we assess whether genomic signatures can be associated with plant parasitism by nematodes. In this review, we report genomic features and characteristics that appear to be common in plant-parasitic nematodes while absent or rare in animal parasites, predators or free-living species. Candidate horizontal acquisitions of parasitism genes have systematically been found in all plant-parasitic species investigated at the sequence level. Presence of peptides that mimic plant hormones also appears to be a trait of plant-parasitic species. Annotations of the few genomes of plant-parasitic nematodes available to date have revealed a set of apparently species-specific genes on every occasion. Effector genes, important for parasitism are frequently found among those species-specific genes, indicating poor overlap. Overall, nematodes appear to have developed convergent genomic solutions to adapt to plant parasitism.

## INTRODUCTION

Nematodes are extremely abundant, speciose and numerically dominate in many ecosystems. Although only 23 000 species have been formally described, estimates of the total number of species range between 0·5 to more than 10 million (Lambshead, 1993; Lambshead and Boucher, 2003; Blaxter, 2011). While the majority of nematodes are free-living species that feed on bacteria or fungi, or that are predators of other nematodes, the phylum Nematoda also includes parasites of vertebrates, invertebrates and plants. These parasites cause damage to animal and human health as well as to agriculture.

Nematodes may have emerged in the Palaeozoic era from a marine habitat during the Cambrian explosion (550–600 million years ago), but due to the extremely scarce fossil record this remains speculative (van Megen *et al.* 2009). Although nematode-like fossils from the Precambrian (Proterozoic era) have been reported (Poinar, 2011), the earliest nematode fossil unequivocally identified to date is from the Palaeozoic era. This ancestral nematode, *Palaeonema phyticum*, has been dated via radiometry to be *c*. 396 million years old (Poinar *et al.* 2008). Interestingly, this nematode was found in stomatal chambers of a fossilized early land plant, *Aglaophyton major*, and may thus represent an early plant-parasitic lineage.

**Fig. 1.**
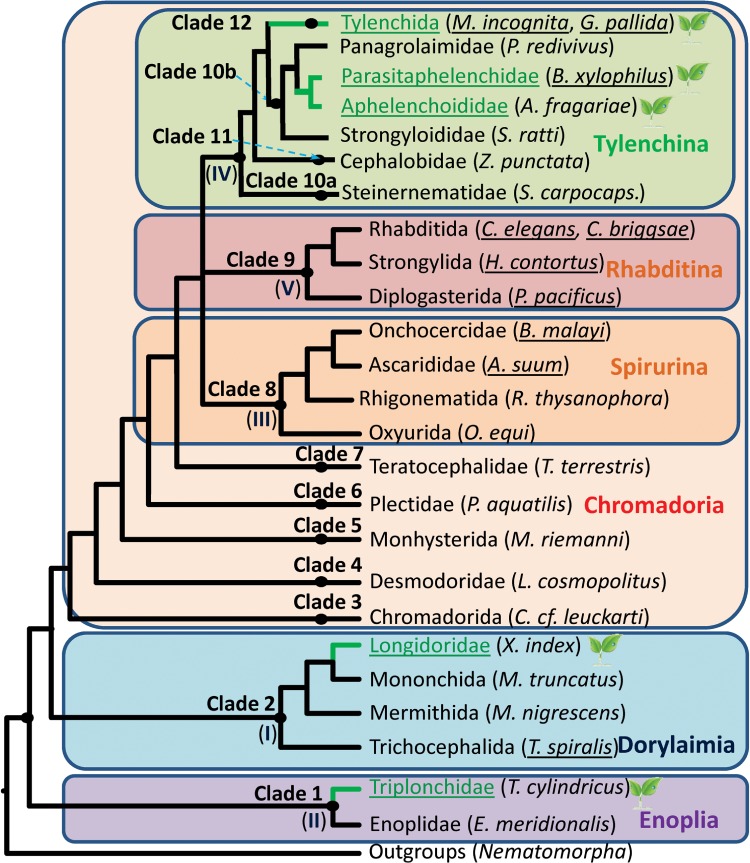
**Schematic phylogeny of Nematoda**. Simplified tree topology modified from van Megen *et al.* (2009), based on SSU rDNA. Clades 1–12 are according to the classification proposed by van Megen *et al.* (2009). Roman numbers I – V correspond to clades that had been defined in Blaxter *et al.* (1998). The three major Nematode lineages Enoplia, Dorylaimia and Chromadoria as described in De Ley (2006) are represented by coloured rectangles. The Chromadoria lineage is further subdivided in Spirurina, Rhabditina and Tylenchina. Taxonomic groups in which plant-parasitic species are found are coloured in green and highlighted by a leaf symbol. Underlined species names indicate availability of a genome assembly. Nematomorpha, a group mainly constituted of parasites of arthropods is the closest outgroup to nematodes.

Resolving nematode phylogeny on the basis of morphological characters is extremely difficult. Few informative characters can be used to distinguish nematode groups and shared morphological features can be the result of convergent evolution as well as homology. However, the availability of molecular markers, such as small subunit ribosomal DNA (SSU rDNA), has allowed significant advances in assessing the phylogeny of nematodes. The most comprehensive phylogeny published to date is based on SSU rDNA sequences from more than 1200 different nematodes (van Megen *et al.* 2009). This phylogeny defines 12 distinct clades within the Nematoda. This classification will be used as a reference in this article (Fig. 1).

### The emergence of plant parasitism in nematodes

The remarkable success of nematodes per se, and parasitic forms in particular, can, at least in part, be attributed to two phylum-wide characteristics. Firstly, nematode development has two distinct phases: (1) embryogenesis, in which a worm is made, and (2) post-embryonic development, in which new structures can be added to the pre-existing animal; vulval-induction in *Caenorhabditis elegans* is the canonical example (Sternberg, 2005). A consequence of this is that the morphological (and other) specializations used in parasitism are potentially free to evolve independent of those genes simply needed to specify the basic nematode body plan. Not surprisingly, parasitic nematodes exhibit a wide range of morphologies. The second phylum-wide attribute of nematodes is their use of secreted proteins to not only define organismal morphology, but also to construct extra-cellular structures, such as the feeding stylet. Together with the developmental plasticity described above, nematodes are thus primed for rapid adaptation to changing environments.

The phylogenetic tree of nematodes shows that plant-parasitic species are not monophyletic. Instead, plant parasitic nematodes are present in at least 4 of the 12 clades defined by van Megen *et al.* (2009) and some of these clades are very distant from one another (Fig. 1). For example, the last common ancestor of the plant-parasitic nematodes from clade 12 (Tylenchida) and from clade 1 (Triplonchidae) is the last common ancestor of all nematodes. In addition, clades containing plant-parasitic nematodes are interspersed by clades comprising free-living species, predators and parasites of animals. The current picture suggests that plant parasitism has arisen at least four times independently in nematodes: within clade 1 Triplonchidae, within clade 2 Longidoridae within clade 10b Aphelenchoididae + Parasitaphelenchidae and within clade 12 Tylenchida (Fig. 1). It is also possible that plant parasitism has evolved on several occasions within some of these clades themselves. For example, within the Aphelenchoididae the majority of species are fungivorous but several plant-parasitic species, including *Aphelenchoides fragariae* and *Aphelenchoides besseyi* are interspersed between the fungivorous species (Rybarczyk-Mydłowska *et al.* 2012). It is unclear whether plant-parasitism was the ancestral state at the base of this clade or whether this lifestyle has emerged independently several times within the clade. Interestingly, clades containing plant parasitic nematodes are frequently closely related to those containing fungivorous nematodes, suggesting that plant parasitism may have evolved from fungal feeding nematodes on each occasion.

Although plant parasitism has evolved repeatedly, all plant-parasitic nematodes have at least one common morphological adaptation, the presence of a syringe-like feeding tool, or stylet, which is used to extract nutrients from plant cells and which, in some species, can also be used to introduce nematode-derived molecules into the host. Although functionally analogous, the precise morphology, ontogeny and structure of this feeding tool varies substantially between clades. The feeding tool is termed the stomatostyle in Tylenchida (clade 12) and Aphelenchoidea (clade 10b), the odontostyle in Longidoridae (clade 2) and the onchiostyle in Triplonchidae (clade 1) (Baldwin *et al.* 2004). The stomatostyle of the Tylenchida and Aphelenchoidea has a lumen that allows nutrients and secretion to pass through but this is not the case for the onchiostlye, where nutrients pass along rather than through the feeding tool. However, while a stylet-like feeding tool seems to be a prerequisite for plant parasitism, several stylet-bearing nematodes are not plant parasites. For example, predators of other nematodes such as *Labronema ferox* (clade 2), blood-sucking animal parasites like *Haemonchus* spp. (clade 9) or fungivorous nematodes like *Aphelenchoides* spp. (clade 10b), all bear a stylet that is used for feeding. Together with the non-homology (in the evolutionary sense) of plant-parasitic nematode stylets, the presence of a stylet in species not associated with plants supports the idea that emergence of this organ is most probably the result of convergent evolution.

Nematodes parasitize plants using a variety of different feeding strategies. At the most basic level, nematodes can be migratory ectoparasites that remain outside the host in the soil and feed on root cells as they are encountered. The majority of species in clade 1 feed using this strategy. Others may be sedentary ectoparasites, which feed for a prolonged period at a single site while remaining outside the roots. Some of these, including several species in clade 2, are also capable of inducing biotrophic feeding structures in their hosts, similar in appearance to the giant cells of root-knot nematodes (below). Endoparasites enter the host and may either be migratory or sedentary. Migratory endoparasites move throughout the plant, causing extensive tissue damage, and feed on the contents of host cells that they encounter. This mode of parasitism is found within clade 10b and clade 12. However, the most damaging species are the sedentary endoparasites. These induce the development of a feeding structure in plant tissues and include the root-knot and cyst nematodes. These sedentary species are responsible for the majority of the estimated ~ € 100 billion damage (McCarter, 2009) caused by nematodes to worldwide agriculture every year.

### Genomes and transcriptomes of plant-parasitic nematodes

The genome sequence of the free-living nematode *C. elegans* was released in 1998 and was the first animal genome sequenced (The *C. elegans* Genome Sequencing Consortium, 1998). It was a further 10 years before the publication of the first plant-parasitic nematode genomes. The genomes of the root-knot nematodes *Meloidogyne incognita* (Abad *et al.* 2008) and *Meloidogyne hapla* (Opperman *et al.* 2008) were also the first genome sequences of any animal that parasitizes plants and offered the first opportunity for comparative genomics between plant-parasitic nematodes. Projects to enhance the assemblies and annotation of both genomes are in progress and for *M. hapla*, those edits are described in the current public freeze (HapPep5). More recently, the genome sequence of the clade 10b species *Bursaphelenchus xylophilus*, has also been published (Kikuchi *et al.* 2011). This nematode is the agent responsible for pine wilt disease and has a complex life cycle that includes beetles as vectors and phases in which the nematode feeds on plant tissues and a fungivorous phase during which the nematode feeds on fungi colonizing dead or dying trees (Jones *et al.* 2008). As a representative of clade 10, it is only distantly related to cyst and root-knot nematodes and, since it is one of the few known plant-parasitic nematodes within the *Bursaphelenchus* genus, probably represents an independent recent evolution of plant-parasitism. No other publication describing the whole genome of a plant-parasitic nematode has yet been released. However, a draft, partially assembled and un-annotated genome for the soybean cyst nematode *Heterodera glycines* is available within publicly accessible databases. This draft genome was produced and patented as a collaborative effort between two companies (patent WO 2007095469). The genome of the potato cyst nematode *Globodera pallida* has recently been sequenced by a consortium of researchers in the UK and a manuscript describing its content is under evaluation at the time of writing (J. T. Jones, personal communication). This genome sequence is publicly accessible through: http://www.sanger.ac.uk/resources/downloads/helminths/globodera-pallida.html. In addition, genome sequences for two clade 12 migratory endoparasites, *Pratylenchus coffeae* and *Radopholus similis*, have recently been sequenced in North Carolina, USA, and a manuscript describing the *P. coffeae* genome has been submitted (C. H. Opperman, personal communication). It is clear that the increased accessibility of high-throughput next-generation sequencing technologies will allow further genome data for a wider range of plant-parasitic nematode species to be obtained in the coming years and that the relatively low costs of obtaining such data will allow nematode species to be selected for sequencing due to their phylogenetic position and biological characteristics rather than just their economic impact. The 959 genomes initiative (Kumar *et al.* 2012) will provide a broader evolutionary framework for these projects.

In addition to the genome sequences described above, substantial expressed sequence tag (EST) resources are available from a range of plant-parasitic nematodes. While these clearly represent partial data that need to be interpreted with some caution, they represent valuable views of the transcriptomes of nematodes. Efforts have been made to organize and assemble EST data from all nematode groups, including plant parasites, and to make the results of these analyses available to the community. For example, NEMBASE is a constantly updated portal to these resources (Parkinson *et al.* 2004; Elsworth *et al.* 2011). Similar resources are available through the nematode.net portal (McCarter *et al.* 2003; Martin *et al.* 2011). The shift from Sanger sequencing to 454 and illumina technologies has boosted the throughput of produced transcriptomic data. For example, the transcriptomes of two root lesion nematodes *P. coffeae* and *Pratylenchus thornei* were published in 2011 (Haegeman *et al.* 2011*a*) and 2012 (Nicol *et al.* 2012), respectively. The transcriptomes of two nematodes whose host range is limited to monocotyledonous plants, *Meloidogyne graminicola*, and *A. besseyi*, a clade 10b species, have also recently been made available (Haegeman *et al.* 2013; Kikuchi *et al.* 2013). The ‘established’ genomes also benefit from higher throughput methods. For example, more than a billion RNA-seq reads were mapped onto the *M. hapla* reference genome (VW9), defining 322 new genes, and correcting the models for 2191 of the 14 420 previously annotated genes (Y.-L. Guo, unpublished data).

## A TREND TOWARDS GENOME REDUCTION IN PLANT PARASITES?

Although it is possible to try to identify genomic adaptations that are characteristic of plant parasitism in nematodes, it is important to note that the genome data generated so far are restricted to the Tylenchida (clade 12) and Aphelenchoidea (clade 10b). Some of the other clades are represented only by EST data and others have not yet been explored at the sequence level. Thus, it is doubtful that observations made on clades 12 and 10b nematodes can be further generalized to all plant-parasitic lineages.

Genome sizes of the plant-parasitic nematodes sequenced to date are smaller than those of adjacent clade 9 species whose genome sequences have been published. With an assembly size of 53 Mb, the *M. hapla* genome is the smallest published so far and about half of the *C. elegans* genome (100 Mb). The migratory endoparasite *P. coffeae* has a genome size of 19·6 Mb as measured by flow cytometry (Leroy *et al.* 2003) and is considered the smallest animal genome. Although the genome sequence of *M. incognita* is larger (86 Mb), this can be largely attributed to a peculiar genome structure, mainly composed of regions in two copies (Castagnone-Sereno *et al.* 2013). Decreased gene sets are also observed in plant parasites. The total number of predicted genes in *M. hapla* (14 040) is much lower than in *C. elegans* (~20 000) and genomic and EST sequence analysis indicates <7500 predicted genes for *P. coffeae* (C. H. Opperman, personal communication). While the clade 10b *B. xylophilus* is bigger both in genome assembly size (74 Mb) and in the number of predicted genes (18 040) than *M. hapla*, it is still substantially smaller than those of clade 9 species. Similarly, the current *G. pallida* genome assembly is in excess of 120 Mb but no more than 16 417 genes are predicted; again fewer than in other sequenced (non-parasitic) nematodes. Although the metrics of *M. hapla, M. incognita, P. coffeae* and *B. xylophilus* are based on draft genomes, while the *C. elegans* genome is completely finished and annotated, the same observation applies to *Pristionchus pacificus*, a clade 9 nematode whose draft genome reaches 169 Mb and that encodes 23 500 predicted genes (Dieterich *et al.* 2008). Thus, although a general rule cannot be directly established from a few genomes, there might be a trend towards genome reduction in plant parasitic nematodes compared with free-living nematode species.

Whether a trend towards genome reduction actually occurs in parasites can be also questioned for animal parasites. The genome of *Trichinella spiralis*, a clade 2 parasite of mammals is only ~64 Mb and contains ~16 000 genes (Mitreva *et al.* 2011). However, the clade 8 filarial nematodes *Brugia malayi, Loa loa* and *Wuchereria bancrofti* all have genome sizes of ~90 Mb, comparable to *C. elegans*, though with fewer predicted genes (~15 000) (Ghedin *et al.* 2007; Desjardins *et al.* 2013). Bucking this trend towards reduction, the genomes of *Ascaris suum* (clade 8) and *Haemonchus contortus* (clade 9) are 273 and ~300 Mb, respectively (Jex *et al.* 2011; Laing *et al.* 2013), which is bigger than those of *C. elegans* or *P. pacificus* and they do not encode substantially fewer genes (18 500 and ~22 000, respectively). Hence, although some examples can be cited, there is no evident general trend for genome reduction in parasitic nematodes.

## HORIZONTAL ACQUISITION OF PARASITISM GENES FROM NON-METAZOAN DONORS

Perhaps the most striking example of a genomic adaptation to plant parasitism is the presence in nematodes of genes that metabolize the plant cell wall. These genes, usually absent in animal genomes, are present in each of the plant parasitic clades examined to date and are thought to have been acquired by horizontal gene transfer (HGT). HGT is the transmission of genes by means other than direct (vertical) inheritance from the parental generation to the offspring. This phenomenon has been widely documented in prokaryotes. For example, pathogenicity factors and genes providing resistance to antibiotics are exchanged horizontally between bacteria regardless of whether the gene is present in the bacterial genome itself or plasmid-borne (Boucher *et al.* 2003; Gogarten and Townsend, 2005). HGT in eukaryotes was previously considered to be a minor evolutionary event, particularly in animals. However, numerous cases of HGT from prokaryotes to eukaryotes have now been reported (Andersson, 2005), including from bacteria to animals (Dunning Hotopp, 2011), suggesting that this phenomenon is more prevalent than originally considered. Some cases of HGT from micro-organisms to animals show a clear link between the transferred genes and a biological function in the receiver organism (Schönknecht *et al*. 2014). The acquisition of genes from bacteria and fungi by plant-parasitic nematodes probably constitutes one of the clearest examples (Danchin, 2011; Haegeman *et al.* 2011*b*; Danchin and Rosso, 2012).

The first report of HGT in plant-parasitic nematodes came with the discovery of cellulase genes in two cyst nematodes, the first cellulase genes in any animal. The absence of these secreted cellulases from any other animal genomes available at that time combined with their highest similarity to bacterial cellulases led to the suggestion that they had been acquired via HGT (Smant *et al.* 1998). The cellulases were subsequently shown to be involved in the degradation of cellulose, one of the major components of the protective plant cell wall, and to play an important role in the invasion of and migration through the host. Similar enzymes were identified in root-knot nematodes soon after (Rosso *et al.* 1999; Ledger *et al.* 2006) and in a range of clade 12 migratory endoparasites, (reviewed in Haegeman *et al.* 2011*b*). A series of other plant cell wall-degrading enzymes were subsequently identified in a variety of plant-parasitic nematodes, including xylanases (Mitreva-Dautova *et al.* 2006), pectate lyases (Popeijus *et al.* 2000; Doyle and Lambert, 2002) and polygalacturonases (Jaubert *et al.* 2002). In addition, expansin-like proteins that disrupt non-covalent bonds in the plant cell wall were also identified (Qin *et al.* 2004). However, in the absence of an available whole genome sequence for a plant-parasitic nematode at that time, the total arsenal of proteins involved in the degradation/softening of the plant cell wall was impossible to assess.

### The repertoire of plant cell wall-degrading enzymes in root-knot nematode genomes

Annotation of Carbohydrate-Active enZymes (CAZymes; Cantarel *et al.* 2009) in the root-knot nematode genomes allowed an assessment of the full repertoire of plant cell wall-degrading enzymes in a plant-parasitic species to be made. *Meloidogyne incognita* was found to harbour 61 putative cell wall-degrading enzymes (Abad *et al.* 2008) and *M. hapla* carries 33 of these (Opperman *et al.* 2008). In addition to the cellulases, xylanases, pectate lyases and polygalacturonases that were already known, examination of the root-knot nematode genomes also identified novel CAZymes including putative arabinanases (GH43 family) and invertases (GH32 family). Invertases are not cell wall-degrading enzymes but cleave sucrose, the major sugar form circulating in plants, into glucose and fructose, which can be readily metabolized by nematodes. Besides these enzymes, expansin-like proteins were also identified in *M. incognita* and *M. hapla*. Analysis of upcoming nematode genomes will soon allow the full repertoire of cell wall-degrading enzymes to be established for additional plant-parasitic nematodes. Representatives of each of these enzyme classes, as well as expansin-like proteins, have already been identified in the genomes and transcriptomes of *P. coffeae* and *R. similis*. However, they occur in greatly reduced numbers, maybe reflecting the different feeding strategies of these migratory endoparasites (C. H. Opperman, personal communication). Both migratory and sedentary endoparasites have to degrade the plant cell wall to penetrate and navigate within plant tissue, but endoparasites also need to modify the plant cell wall for establishment of a feeding site.

**Fig. 2.**
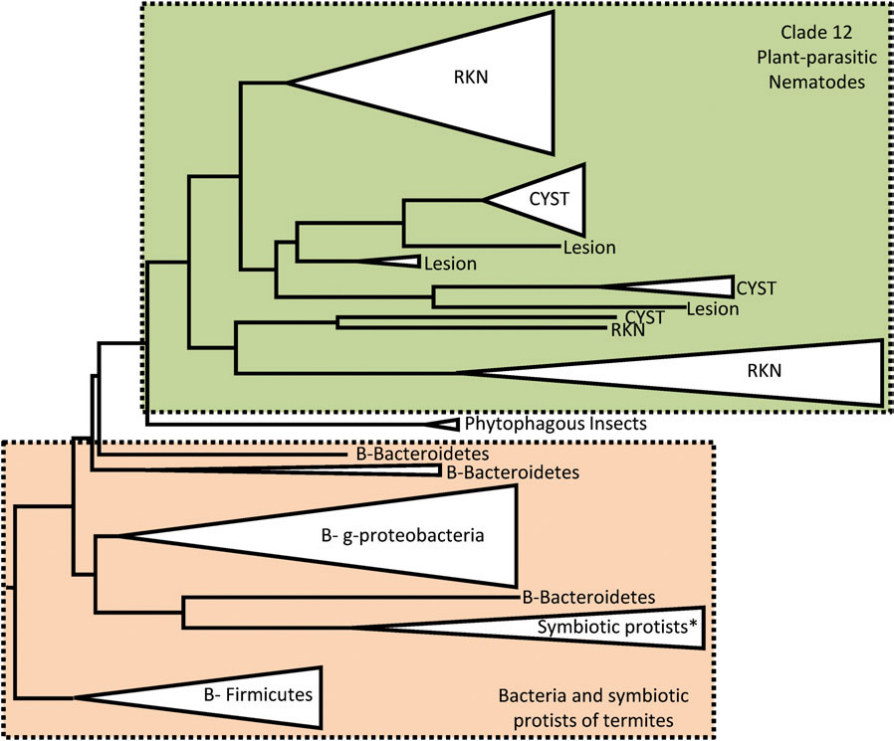
Phylogenetic relations of nematode GH5 cellulases. This simplified phylogenetic tree is adapted from Danchin *et al.* (2010) and represents the evolutionary relations between nematode GH5 cellulases and their closest homologues in other species. RKN stands for ‘root-knot nematodes’, CYST for ‘cyst nematodes’, Lesion for lesion nematodes (*Radopholus similis*), B for bacteria. The phytophagous insects represented in this phylogeny are *Apriona germari* and *Psacothea hilaris*.

Although BLAST-based similarity searching suggested that the genes encoding cell wall degrading and modifying enzymes had been acquired via HGT, only a precise phylogenetic analysis allows the evolutionary history of these proteins to be determined. To address this, a systematic phylogenetic analysis was conducted for each of these proteins, using the root-knot nematode genomes as queries (Danchin *et al.* 2010). Xylanases, pectate lyases, polyglacturonases, candidate arabinases and expansin-like proteins all showed highest similarities to bacterial or fungal proteins. The tree topologies indicating closest homology to microbial proteins were highly supported and their likelihoods were significantly higher than those of alternative topologies. The only exception was for cellulases. Here, the tree topology showed closest homology to cellulases of two herbivorous insects then immediately after, to bacteria (Fig. 2). One possibility is that cellulase genes from bacteria not yet sampled in the sequence database have been transferred twice, once to plant-parasitic nematodes and on another occasion to these herbivorous insects. The absence of cellulase in the many insects in sequence databases argues in favour of this HGT to insects. However, whether these cellulases are ancestral in insects or have been acquired via HGT is still the subject of debate (Watanabe and Tokuda, 2010).

### HGT in other nematode clades

There is evidence suggesting that genes encoding cell wall-degrading enzymes are also present in other clades of plant parasitic nematodes. The most striking example is provided by the presence of GH45 cellulases in clade 10b plant-parasitic nematodes (Aphelenchoidea). Unlike GH5 cellulases, which resemble bacterial enzymes, these GH45 cellulases, described from *Bursaphelenchus* (Kikuchi *et al.* 2004) and *A. besseyi* (Kikuchi *et al.* 2013) are most similar to fungal cellulases and it seems likely that they have been acquired by HGT from fungi. The genome sequence of *B. xylophilus* revealed that this species harbours 11 GH45 genes but no GH5 gene (Kikuchi *et al.* 2011). A wider screen of GH45 genes in nematodes and fungi showed nematode GH45 genes have close phylogenetic relationships and conserved gene structure with fungal homologues (T. Kikuchi, unpublished data). Hence, the acquisition of cellulases, while representing a trait common to several clades, is probably the result of convergent and independent evolution. By contrast, families such as the PL3 pectate lyases and expansin-like proteins are found both in clade 10b and clade 12 nematodes and whether they have been acquired in an ancestor of the two clades or twice, independently, remains to be established.

In addition, an entirely different class of cellulases (GH12) may also be present in a clade 2 plant-parasitic nematode, *Xiphinema index* (Jones *et al.* 2005); E. G. J. Danchin, J. T. Jones & J. Helder, unpublished data). The small size and difficulty of obtaining large quantities of clade 1 nematodes means that no molecular analysis of these nematodes has been carried out to date. Whether cell wall-degrading enzymes are also present in this clade is therefore unknown. However, the data available to date suggest that the ability to metabolize the plant cell wall is indeed a common genomic adaptation required for plant-parasitism. It can be hypothesized that HGT has provided the nematodes with new abilities that certainly allowed them to access a new ecological niche. This probable selective advantage associated with the transfer probably was responsible for successful fixation of the foreign gene at the level of populations then of the species itself (Danchin, 2011).

### The importance of HGT in the plant-parasitic ability of nematodes

The plant cell wall-degrading enzymes provide a clear example of acquisitions via HGT in the genome of a plant-parasitic nematode with evident roles in plant parasitism. However, the total contribution of foreign genes to plant-parasitic capacity in nematodes might not be restricted to the degradation of the plant cell wall. An analysis of all the reported cases of HGT in plant-parasitic nematodes from the literature showed that HGT may be associated with processes other than degradation of the plant cell wall (Haegeman *et al.* 2011*b*). Genes acquired by HGT may have also contributed to other important processes supporting plant parasitism, including suppression of host defences (e.g. cyanate lyases and chrorismate mutases), nutrient processing (e.g. candidate invertase and enzymes from the salvage pathway of vitamins B1, B5, B6 and B7) or establishment of a feeding structure (e.g. NodL like). A recent systematic search for HGT has shown that, in total, more than 3% of protein-coding genes in root-knot nematodes may have been acquired from non-animal donors (Paganini *et al.* 2012).

## OTHER NEMATODE PROTEINS REQUIRED FOR INTERACTIONS WITH THE HOST – EFFECTORS

All nematodes that parasitize plants are faced with similar obstacles to obtaining food. As discussed above, each will need to overcome the barrier presented by the host cell wall. All nematodes will also need, to some extent, to overcome or suppress host defence responses although this is clearly of greater importance to endoparasitic species and to those that induce a biotrophic feeding structure than it is to migratory ectoparasites. Indeed, some species need to induce a feeding structure, and these show a wide range of forms and ontogenies. Remarkably, the ability to induce these feeding structures seems to have evolved independently in several clades, including clade 2 (*Xiphinema* and *Longidorus* species) and clade 12. Within clade 12, this ability has evolved independently in the cyst nematodes (which induce a syncytium) and the root knot nematodes (which induce giant cells). The nematode molecules that mediate these interactions are termed effectors. Various definitions for effectors are used by different authors but for the purposes of this review we consider effectors as nematode molecules that suppress host defences or manipulate the host to allow provision of food to the nematode.

Analysis of the effectors present in various nematode groups shows that they reflect the independent origin of biotrophy in nematodes. For example, a comparison of the effectors present in root-knot and cyst nematodes showed that there are almost no effectors shared between these species (J. T. Jones, personal communication). Supporting these views, ESTs derived from both root-knot nematodes and cyst nematodes show that effector sequences include a very high proportion of pioneer sequences that are not found outside the genus (Gao *et al.* 2003; Huang *et al.* 2003). Some of these have evolved to perform essential and highly specific functions. For example, the ‘19C07’ effector from cyst nematodes, which has no similarity to other sequences in the databases, has been shown to interact with an auxin influx transporter (LAX3) and may increase LAX3-mediated auxin influx in order to promote syncytium development (Lee *et al.* 2011).

There are also clear examples of root-knot and cyst nematodes using different molecular strategies to achieve the same functional goal. For example, a secreted calreticulin is used by *M. incognita* to suppress host defence responses (Jaouannet *et al.* 2013). Although calreticulins are also present in cyst nematodes, there is no evidence to show that these are deployed as effectors. A variety of other molecules including secreted SPRY domain proteins (Postma *et al.* 2012) and ubiquitin extension proteins (Chronis *et al.* 2013) fulfil this functional role in cyst nematodes. One feature of effector families in *G. pallida* is that in many cases they are present in large multigene families. The most striking example of this is provided by the SPRYSEC gene family which consists of almost 300 different genes in *G. pallida* but other effector gene families are also expanded (J. T. Jones, personal communication). Since effectors (or their activity) are recognized by host resistance proteins, this expansion of effector gene families is likely to result from selection pressure to evade recognition by the host. Consistent with this, one *G. pallida* SPRYSEC effector has been identified as an avirulence gene, and is recognized by the Gpa2 resistance plant gene (Sacco *et al.* 2009). Expansion of effector gene families does not seem to be present at a comparable amplitude in the genomes of root-knot nematodes, and whether this represents a common adaptation to plant parasitism remains to be clarified.

## NEMATODE-ENCODED PLANT PEPTIDE HORMONES: A SPECIAL CLASS OF EFFECTOR?

Many processes in vascular plants are controlled by peptide hormones. These proteins, typically 12–15 amino acids long, serve as secreted ligands to mediate cell-to cell communication. The canonical exemplar is the CLAVATA system, in which a peptide (generically termed ‘CLE’) is secreted into the apoplast to be perceived by transmembrane receptors on adjacent or nearby cells. Arabidopsis encodes at least 600 proteins that fall into this class of receptor, termed Receptor–Like Kinases (RLK), as well as 32 potential ligands. Because the RLK may function as hetero-dimers, the permutations of receptor-ligand complexes is large, presumably affording a high degree of functional plasticity to these regulatory circuits. Genetic evidence implicates components of these circuits as playing a role in nematode parasitism. For example, plants carrying mutations in a well-characterized CLE receptor, the *har-1* locus in *Lotus japonicus* (Sharma *et al.* 2003; Hirakawa *et al.* 2008), are hyper-infected by RKN (Lohar and Bird, 2003). Independently, it has been found that polymorphisms in the orthologous locus in tomato correlate with root-knot nematode virulence (Ejima *et al.* 2011). Collectively, these findings are consistent with the broader hypothesis that root-knot nematodes effect developmental changes necessary for feeding site formation/function, at least in part, by secreting analogues of plant peptide hormones (Bird, 1996).

This hypothesis was first supported by a computational screen (Olsen and Skriver, 2003) that revealed that the SYV-46 peptide from soybean cyst nematode (SCN: *H. glycines*) likely encoded a CLE-like ligand. SYV-46 had previously been experimentally identified as a protein secreted from the SCN stylet (Gao *et al.* 2003). Not only does the SYV-46 protein bind a bona fide CLE receptor, CLV2, but the *syv-46* gene also is able to complement the Arabidopsis *clv3-1* mutant (Wang *et al.* 2005), strongly implicating this peptide as being a genuine CLE. The *syv-46* gene appears to have undergone a recent duplication, as the SCN genome contains a second copy, differing by just 3 bases outside the CLE domain (Davis *et al.* 2008; Mitchum *et al.* 2008; Lu *et al.* 2009). Like plant-encoded CLE, the SCN-encoded CLE mimics contain an additional ‘pro’ domain between the signal sequence and active peptide. For the native CLE, cleavage of the pro-domain from the active peptide occurs in the apoplast (Ni *et al.* 2011), and presumably serves an additional regulatory function. Recent data confirm the presence of SCN secretions within host cytoplasm, and the variable domain within HgCLE has been implicated in the transportation of HgCLE from the host cytoplasm to the apoplast, the presumed site of action. Fusion proteins were constructed by switching the variable domains of native CLE and HgCLE. Based on phenotypic analysis of root development, the variable domains between HgCLE and native CLE are reported as interchangeable, despite the seemingly contradictory functions (Wang *et al.* 2010). GFP-tagged antibodies raised against HgCLE possibly indicate localization to syncytia cytoplasm (i.e. cyst nematode feeding sites). In the same report, evidence derived from the transient over-expression of protein fusions in plants and subsequent bioassays indicates an apoplastic destination.

The role of CLE in the interaction between root-knot nematodes and their hosts has been less studied. Like the *H. glycines* SYV-46 protein, an *M. incognita* protein called 16D10 was initially isolated as an anonymous, putatively secreted protein (Huang *et al.* 2003) and later computationally identified as having sequence similarity to the CLE motif (Huang *et al.* 2006). Transgenic over-expression of 16D10 gave a root developmental response and it was found that the nematode ligand bound to two host SCARECROW-LIKE (SCL) proteins; this result was consistent with the result from a yeast 2-hybrid assay (Huang *et al.* 2006). SCL are members of the GRAS class of transcription regulators, which play central roles in root meristem specification and also are central to rhizobial nodulation (Hirsch *et al.* 2009), a process with many molecular and developmental similarities to giant cell induction (Bird, 2004; Weerasinghe *et al.* 2005). The rather surprising but robust finding of 16D10 binding to the nuclear protein SCL, rather than a trans-membrane receptor in the apoplast, appears to dismiss this particular gene as encoding a CLE (Mitchum *et al.* 2008).

**Fig. 3.**
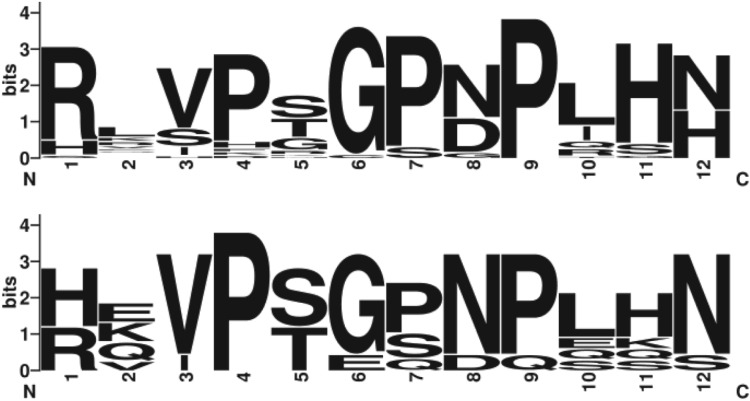
Nematode CLE motifs. LogoPlots of the 27 unique CLE domains in Arabidopsis (top) and the nine unique candidate CLE domains from *M. hapla* (bottom). Each *M. hapla* domain was included four times to balance the amplitude.

Interrogation of the assembled genomes using a double-affine Smith-Waterman algorithm revealed numerous candidate CLE loci in root-knot nematodes (Fig. 3), including many likely false positives. Adding the additional requirements of encoding a secretion signal sequence and a predicted cleavage site upstream of the conserved carboxyl-terminal domain limits the number of CLE in *M. hapla* to eight genes (D. McK. Bird, personal communication). Each is predicted to encode just these two domains. Absence of the ‘pro’ domain from the mimics encoded by root-knot nematodes is consistent with the injection of the active form of the peptide hormone directly into the apoplast where they presumably interact with host RLK and circumvent host regulation. Lesion nematodes, which do not form feeding sites, lack detectable CLE. Functional analyses define two classes, ‘A’ and ‘B’. A-type CLE, which include CLV3, promote cell differentiation at the meristem by antagonizing *WUS* and aborting root growth. B-type CLE do not promote cell differentiation at the meristem, but inhibit cell differentiation in *Zinnia elegans* xylem elements. Members of the different CLE classes likely interact in various manners. Based on sequence similarity, *M. hapla* and *M. incognita* encode both types of CLE seen in *Arabidopsis*.

### Root-knot nematode-encoded CEP mimics

**Fig. 4.**
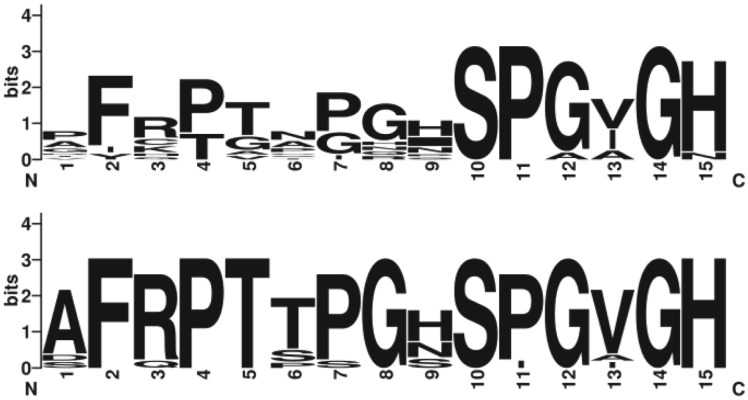
*Meloidogyne hapla* CEP domains. LogoPlots of the 12 CEP domains from *M. hapla* (top) aligned with the 11 unique CEP from *Medicago truncatula* (bottom).

It is assumed that canonical plant peptide hormones are encoded by multiple paralogous genes, with relatively small products ranging from 70–110 amino acids, lacking notable secondary structure (such as cysteine residues) and with high sequence diversity except for the c-terminal domain (Ohyama *et al.* 2008). Using an algorithm based on these assumptions, a novel, multi-member class of 15-amino acid plant peptide hormones, collectively known as CEP (C-terminally Encoded Peptide) were computationally identified. CEP are expressed in lateral root primordia, and based on the presence of a signal sequence and mass spectrometry data, are postulated to be hormone ligands. The inhibition of lateral root development by transgenic over-expression and the phenocopying effect of exogenous application of synthetic CEP peptide indicate an apoplastic manner of action, congruent with a peptide hormone. In the original report (Ohyama *et al.* 2008), five CEP were revealed, but our data show an additional protein with five CEP motifs and another with two motifs can be found in the Arabidopsis genome (D. McK. Bird, personal communication). Consistent with a role in regulating lateral root development, CEP are widely distributed across vascular plants, but appear absent from moss or unicellular green algae. Interrogation of the *M. incognita* and *M. hapla* genomes revealed 8 and 12 CEP genes, respectively (Fig. 4). CEP are not found in any other animal genera, including cyst and lesion nematodes. Like their plant analogues, each root-knot nematode gene encodes a signal sequence at the amino terminus and single CEP motif at the carboxyl terminus. As is the case for the CLE, plant CEP include a domain between the signal sequence and the hormone domain, which most likely represents a pro-protein domain that is proteolytically removed in the apoplast. Like root-knot nematode CLE, the CEP mimics lack this domain, presumably allowing for the direct delivery of an active peptide into the apoplast.

### CLE and CEP loci

**Fig. 5.**
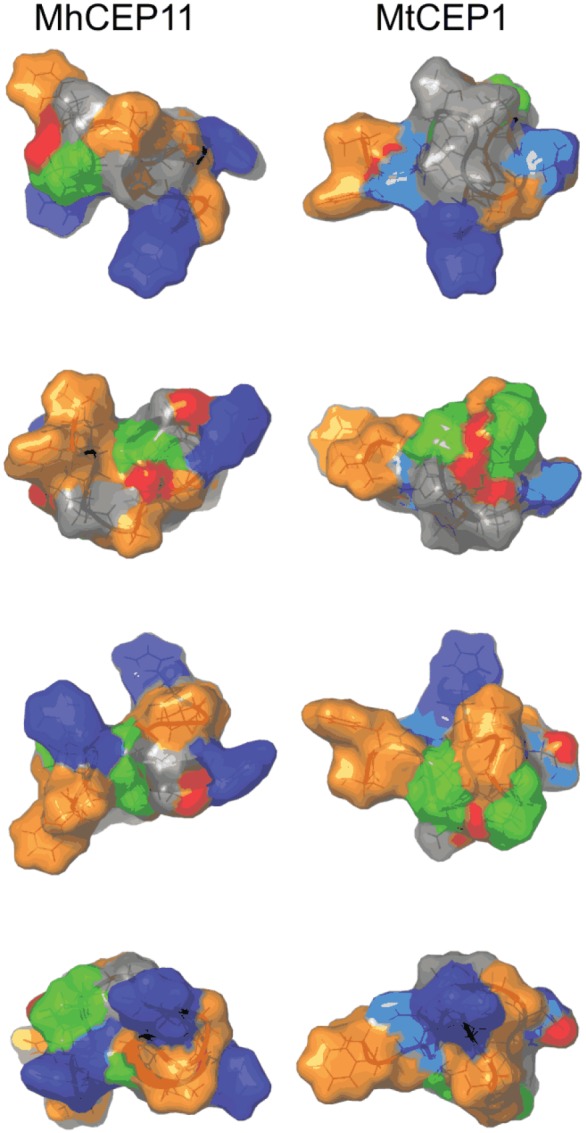
Comparison of surface characteristics of nematode and plant CEP domains. Surface characteristics of CEP11 from *Meloidogyne hapla* (MhCEP11) and *Medicago truncatula* CEP1 (MtCEP1), shown through a 90° rotation series. Residues are colour-coded by physico-chemical property: Orange  =  hydrophobic; Blue  =  positive residues; Cyan  =  Asp; Green  =  Pro; Red = Hydroxyl group on Pro.

The evolutionary origin of nematode-encoded, plant peptide hormone mimics remains unclear. The notion that they were acquired from their plant hosts (by HGT) is appealing, but the evidence necessary for such inference (i.e. phylogenetic incongruence between species and gene trees) is lacking. Because they encode short sequences, CEP and CLE loci inherently have a restricted phylogenetic signal. This is exacerbated by the functional constraints of the signal sequence and an active hormone domain. Thus, although correspondence can be established between each root-knot nematode CLE (or CEP) and an analogous gene in Arabidopsis, this likely represents functional equivalence rather than reflecting evolutionary homology. We stress however that although evidence for evolutionary homology is lacking, it cannot be ruled out that these gene families arose from a common ancestor (homology) which may have been in a different kingdom (HGT). Nonetheless it has been proposed that these nematode proteins may have arisen *de novo* (i.e. convergently: Sikora *et al.* 2005; Mitchum *et al.* 2008) rather than by HGT and circumstantial evidence, at least for the CEP, may support this. In *M. hapla*, the 12 CEP-encoding genes are grouped into two tightly linked clusters on a single chromosome. Routine annotation of these regions, using gene-finding algorithms, trained on large EST datasets, failed to identify any genes in these areas where CEP have now been found. Such gene-sparse regions are highly atypical within the *M. hapla* genome and unlikely to be coincidental. Comparison of the two CEP loci between sequenced *M. hapla* isolates (VW8 and VW9) indicate these regions are hypervariable. Collectively, we hypothesize that these are areas of the genome exhibiting rapid evolution, presumably reflecting high diversifying pressure, which in itself may be expanding CEP functions in the parasitic interaction. By contrast CLE, which based on their presence in cyst nematodes as well as root-knot nematodes might to be evolutionarily more ancient, are not surprisingly distributed throughout the root-knot nematode genome. Consistent with expanded function, molecular dynamics simulations of the NMR-solved structures of plant and nematode-encoded CEP (Bobay *et al.* 2013) indicated that the root-knot nematode ligands have the potential to sample more conformational space than the endogenous peptides (Fig. 5). Perhaps this feature also contributes to the broad host range of these nematodes.

## PROTECTION BY THE HOST – PLANT PARASITIC NEMATODES HAVE REDUCED IMMUNE SYSTEMS

One genomic consequence of the capacity to reside within a plant seems to be the ability to shed a significant number of genes involved in defence against pathogens. In root-knot nematode genomes, a reduction in the repertoire of genes involved in detoxification, immunity and defence against bacteria and fungi is observed. For example, a very reduced set of chitinases is present as compared with other nematodes as well as a simplified glutathione S transferase gene family, compared with that present in *C. elegans* (Abad *et al.* 2008). A reduced set of chitinases is also present in *M. hapla* (Opperman *et al.* 2008). Chitinases are considered as antifungal proteins in nematodes and GSTs are used for detoxification. The genome of *G. pallida* contains fewer genes involved in detoxification of secondary metabolites as compared with *C. elegans* and genes encoding immune effectors (lysozymes, C-type lectins and chitinases) are also reduced in number (J. T. Jones, personal communication). Is this a feature common to all plant-parasitic nematodes or common to sedentary endoparasites only? First insights into the genomes of *P. coffeae* and *R. similis* also show a reduction of these families involved in defence and immunity (C. H. Opperman, personal communication). These data suggest, paradoxically, that although plant-parasitic nematodes need to avoid or suppress host defence responses, they also derive significant benefit from these host processes in that they are protected from soil-dwelling pathogens while inside their host. The impact of this protection can be clearly seen in the genomes of some plant-parasites. However, it should also be noted that this is not a constant feature of plant-parasitism. The genome of *B. xylophilus* seems to have an expanded repertoire of genes encoding proteins involved in detoxification processes – this may reflect the fact that this nematode needs to inhabit a plant and an insect at various phases of the life cycle or it may reflect the specific challenges of surviving in woody tissues, which are particularly rich in secondary metabolites (Kikuchi *et al.* 2011).

Interestingly, a reduction in the set of antibacterial genes was observed in the clade 8 animal-parasitic nematodes *L. loa, B. malayi* and *W. bancrofti* (Desjardins *et al.* 2013). However, such a reduction of antibacterial repertoire was not observed in *A. suum*, suggesting this might be a specificity of the above-mentioned filarial nematodes. Further supporting this filarial specificity, no reduction of antibacterial, detoxification or immune repertoires have been mentioned in the genome papers of the animal parasites *T. spiralis* (Mitreva *et al.* 2011) and *H. contortus* (Laing *et al.* 2013).

## MODES OF REPRODUCTION – THE INFLUENCE OF HOST RANGE AND THE EFFECTS ON NEMATODE GENOMES

The host range of plant-parasitic nematodes has an enormous influence on their biology, including their mode of reproduction (Blok *et al.* 2008) and this, in turn may have a significant impact on genome structure. Most plant pathogens, including nematodes, are unable to develop on most plants and therefore have a restricted host range. Many cyst nematodes follow this rule, with each species able to infect a relatively narrow range of plants. The host is often reflected in species names (potato cyst nematode, soybean cyst nematode and so on). As a consequence, a cyst nematode developing on a host is likely to encounter another individual of the same species on that host and reproduction is most often sexual in these species. However, some root knot nematodes, including *M. incognita* have extraordinarily broad host ranges. Although the whole host range of *M. incognita*, as a species, might be the result of an assemblage of populations that have each a more restricted collection of compatible hosts, most tested populations have multiple host plants (Robertson *et al.* 2006; Castagnone-Sereno *et al.* 2013). Root-knot nematodes show a striking diversity of reproductive modes, ranging from strict sexual reproduction (amphimixis), facultative sexual reproduction (meiotic parthenogenesis) to fully asexual reproduction (mitotic parthenogenesis). Interestingly, root-knot nematodes with the broadest host ranges, like *M. incognita*, reproduce by mitotic parthenogenesis, which ensures that reproduction can occur in the absence of a partner. This reproductive mode does not require finding a mate and allows a rapid and effective exploitation of food resources. These observations suggest that there is a link between the mode of reproduction and the host range. The mode of reproduction itself has consequences for the genome structure. Absence of meiosis relaxes pressure for homologous chromosome pairing and allows them to substantially diverge from one another. Supporting this view, the genome of *M. incognita* is present in pairs of regions showing a high average divergence of 7% at the nucleotide level. It is hypothesized that this high divergence might allow functional divergence between gene copies thus providing a mechanism of plasticity/variability in the absence of inter-individual genetic exchanges. Whether these genomic singularities are common in strict parthenogenetic plant-parasitic nematodes will be interesting to assess as new genomes become available.

A point that is common to cyst nematodes and root-knot nematodes is sex determination. In both groups of species, sex determination depends on environmental conditions with adverse conditions leading to more males being produced in both cases. In facultative parthenogenetic species, it is hypothesized that the higher proportion of males generated during adverse conditions allows for more sexual reproduction to occur. Promoting genetic exchange between individuals, sexual reproduction would allow new combinations of alleles to emerge, with enhanced probability of including new genotypes able to adapt. Here again, while the genetics underlying this process is not described it is possible that this mode of sex determination, determined by the environment, has emerged independently in a convergent manner.

## CONCLUSIONS

Plant parasitic nematodes can collectively infest virtually any human-cultivated plant and cause dramatic damage to worldwide agriculture. They can therefore be considered as evolutionarily successful parasites. This feeding mode has emerged several times independently in nematodes. Interestingly, the challenges constituted by infesting a live host plant appear to have led to parallel convergent evolution of similar solutions. For instance, at a morphological level, all plant-parasitic nematodes possess a syringe-like stylet to feed from plant cells. This organ appears to have been invented independently in each clade with different ontogeny and fine structure. Exploring recent genomic data, we have shown here that, just like observations at the morphologic level, convergent genomic adaptations also appear to have occurred independently in different lineages of plant-parasitic nematodes. Acquisition of parasitism genes via HGT appear to be an adaptation common to all species investigated at the sequence level. Effector proteins, involved in repression of plant defence systems and establishment of a successful biotrophic interaction are encoded by the genomes of various plant-parasitic nematodes. However, these effector proteins are poorly conserved from one nematode species to another and mostly lineage-specific, suggesting that they also have emerged independently multiple times to circumvent similar plant host challenges. Mimicry of plant peptide hormones is a common feature of cyst nematodes and root-knot nematodes, two independently evolved lineages of sedentary endoparasitic nematodes. Interestingly, there is no evident orthological relationship between cyst and root-knot nematode mimics, indicating, once again, a probable convergent evolution. Overall, while some features, such as the acquisition of parasitism genes via HGT, reduction of the repertoire of genes for detoxification and defence, the presence of effector genes or of plant peptide mimics appear as common genomic signatures, looking into each of these features in more detail reveals lack of true homology and suggests independent emergence of convergent adaptations to plant parasitism within the different nematode lineages.

Interestingly, several parallels can be drawn between the genomes of plant-parasitic nematodes and those of other phytopathogens or phytoparasites. For instance, acquisition via HGT of plant cell wall-degrading enzymes and other parasitism genes have also been observed in plant-parasitic oomycetes (Judelson, 2012). Similarly, phytoparasitic fungi have acquired genes involved in pathogenesis from bacteria via HGT too (Gardiner *et al.* 2012). Filamentous fungi as well as oomycetes also encode genus or lineage-specific effectors secreted *in planta* that assist different processes enabling parasitism (Oliva *et al.* 2010). Hence, these two features appear to represent more general adaptive convergences to plant parasitism.
